# Asymmetric bilayer dressings with spatiotemporal sequence loaded with IL‐24 and GCDs for the treatment of diabetic wounds

**DOI:** 10.1002/ctm2.70402

**Published:** 2025-07-16

**Authors:** Sijia Li, Jinjin Lu, Nianqiang Jin, Yuan Su, Songning Han, Jiankang He, Wenqiang Xie

**Affiliations:** ^1^ Foshan Stomatology Hospital & School of Medicine Foshan University Foshan P. R. China; ^2^ Department of Periodontics Affiliated Stomatology Hospital of Guangzhou Medical University, Guangzhou Key Laboratory of Basic and Applied Research of Oral Regenerative Medicine Guangzhou P. R. China; ^3^ Department of Prosthodontics Stomatological Hospital, Southern Medical University Guangzhou PR China; ^4^ Stomatology Center, Shunde Hospital, Southern Medical University (The First People's Hospital of Shunde) Foshan P. R. China; ^5^ School of Stomatology Jinan University Guangzhou P. R. China

1

Dear Editor

Diabetic wounds pose a significant global health challenge, affecting hundreds of millions of diabetes patients and imposing substantial financial burdens on healthcare systems.^1^ Despite conventional treatments, the underlying mechanisms remain poorly understood, resulting in unsatisfactory clinical outcomes. Diabetic wounds exhibit severely impaired fibroblast‐to‐myofibroblast transition (FMT), a process essential for wound contraction and matrix remodelling. Hyperglycaemia, advanced glycation end products, and chronic inflammation disrupt key signalling pathways regulating FMT, resulting in deficient myofibroblast differentiation and compromised wound healing.^2^ Recent biomaterial advances in diabetic wound care include hydrogel dressings incorporating growth factors, cytokines, and antimicrobial agents. However, current approaches typically target single mechanisms and fail to simultaneously address impaired cellular function, bacterial infection, and chronic inflammation—the complex interplay characterising diabetic wounds.^3^ Interleukin‐24 (IL‐24) exhibits exceptional promise in stimulating cellular proliferation, differentiation, and extracellular matrix production.^4^ We postulate that IL‐24 may facilitate the impaired FMT, thereby enhancing diabetic wound repair. Nevertheless, clinical application encounters significant obstacles, including potential bacterial attraction and challenges in maintaining stable, sustained local delivery at wound sites.^5^ To address IL‐24′s bacterial chemotaxis, we incorporated ginseng‐derived carbon quantum dots (GCDs) possessing wide‐ranging antimicrobial effects and superior biocompatibility.^6^ In this study, we discovered significant IL‐24 upregulation during diabetic wound healing. Using IL‐24 knockout mice, we demonstrated that IL‐24 promotes healing by inducing FMT through in vivo and in vitro experiments. Building on these findings, we developed a novel asymmetric dual‐layer hydrogel dressing. The tissue side layer delivers IL‐24 to enhance FMT, while the outside layer, containing GCDs, provides antibacterial effects through reactive oxygen species (ROS) release. Animal models confirmed this dressing's dual efficacy in promoting wound healing and preventing infection. Our findings establish IL‐24 as a promising therapeutic target and demonstrate that this dual‐layer hydrogel represents an innovative strategy for diabetic wound management.

Analysis of gene expression signatures from GEO databases revealed significant IL‐24 upregulation in diabetic wounds, demonstrating expression patterns parallel to established wound healing genes (Figure 1A–C). We confirmed these bioinformatic findings through immunohistochemical (IHC) analysis of STZ‐induced diabetic C57 mice, which validated elevated IL‐24 expression in diabetic wound tissues (Figure 1D–F). To elucidate IL‐24′s functional significance, we generated IL‐24 knockout (KO) mice and established a diabetic model with dorsal wounds (Figure 1G and H). Planimetric assessment demonstrated that IL‐24^KO^ diabetic mice exhibited markedly impaired wound closure (merely 60% by day 6) compared to wild‐type diabetic controls (80% closure) (Figure 1I–K). Histological examination through H&E staining corroborated these findings, revealing substantially wider wounds in IL‐24 ^KO^ diabetic mice (Figure 1L and M). Masson staining identified loosely arranged collagenous architecture and significantly diminished fibrosis ratios in IL‐24^KO^ diabetic wounds, indicating compromised fibroblast function during the proliferative healing phase (Figure 1N and O). Notably, myofibroblast populations characterised by α‐SMA expression were considerably reduced in IL‐24^KO^ diabetic wounds, suggesting dysregulated FMT. Immunofluorescence (IF) analysis revealed attenuated proliferative capacity of myofibroblasts (Ki67+/Vimentin+) in IL‐24^KO^ specimens (Figure 1P and Q), while IHC confirmed decreased α‐SMA and COL1 expression, indicating impaired extracellular matrix production (Figure 1R–U). Collectively, these findings demonstrate that IL‐24 deficiency significantly compromises wound healing progression in type 1 diabetic mice through myofibroblast dysfunction.

**FIGURE 1:**
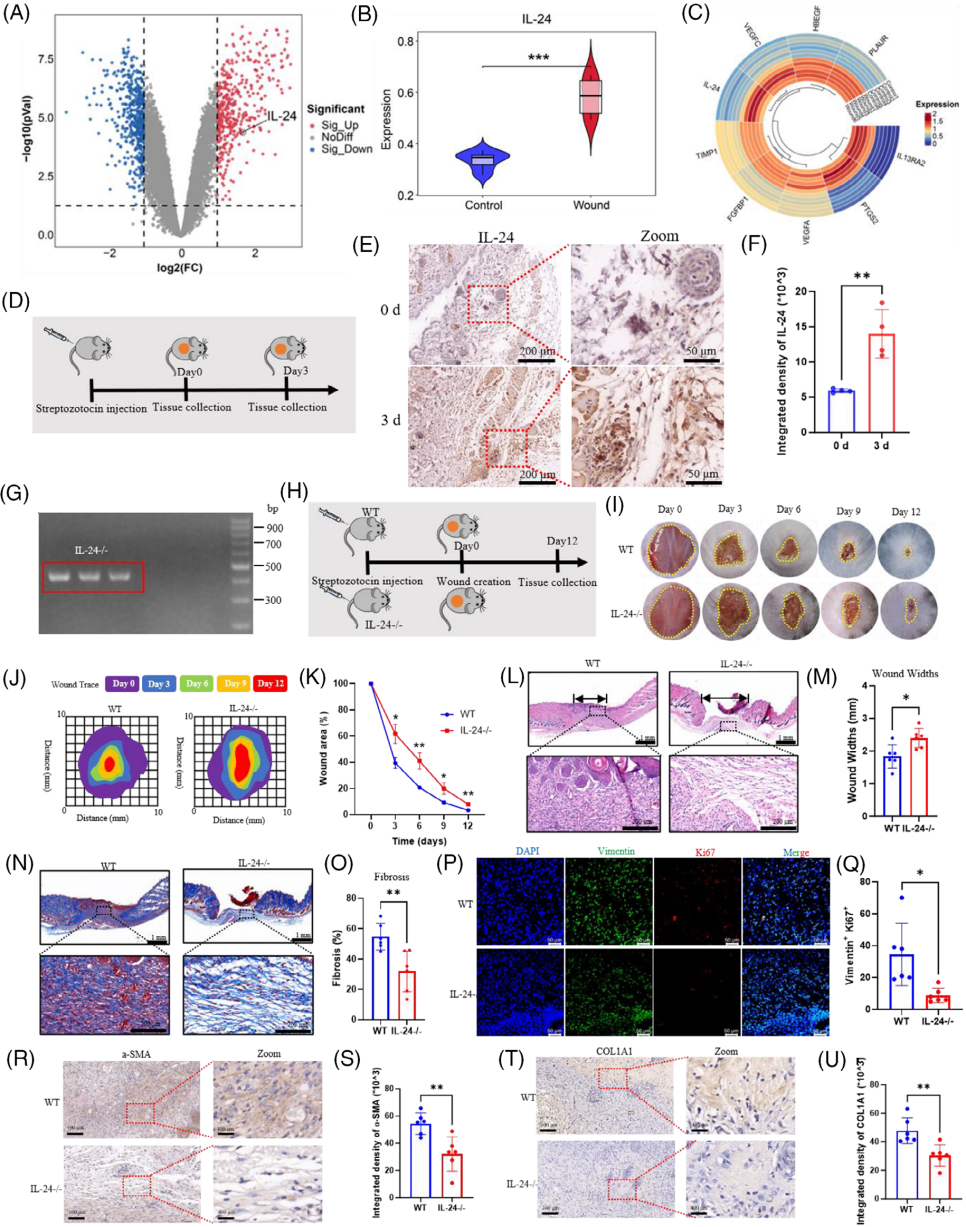
Analysis results of differentially expressed genes (IL‐24) in diabetic wounds, including (A) volcano plot, (B) violin plot and (C) heatmap. (D) Schematic diagram of sampling from diabetic wounds on day 0 and day 3. (E, F) IHC staining showing the expression of IL‐24. (*n* = 4 per group.) (G) The genotyping result of IL‐24^KO^ (IL‐24^–/–^) mice. (H) Schematic diagram of the diabetic wound construction of IL‐24^–/–^ mice and WT mice. (I) Images showing morphological changes of the diabetic wounds. (J) The wound trace of the diabetic wounds. (K) The wound area ratio of the diabetic wounds. (*n* = 6 per group.) (L, M) H&E staining showing changes of the diabetic wounds. (*n* = 6 per group.) (N, O) Masson staining showing the changes of fibrosis area. (*n* = 6 per group.) (P, Q) IF staining revealing the expression of Vimentin and Ki67 in the diabetic wounds. (*n* = 6 per group.) (R, S) The expression of α‐SMA was demonstrated by IHC staining. (*n* = 6 per group.) (T, U) IHC analysis depicted the COL1A1 expression. (*n* = 6 per group.) *p < .05, **p <.01, one‐way ANOVA followed by Bonferroni's multiple comparison test. All values are presented as means ± SEM.

To investigate IL‐24′s impact on fibroblasts, we employed L929 cells as an in vitro model. CCK‐8 assays identified 100 ng/mL as the optimal concentration for enhancing proliferation (Figure 2A). Previous studies have shown that the in vivo levels of IL‐24 range from pg/mL to ng/mL levels. Our 100 ng/mL concentration is well‐established in literature, with studies demonstrating optimal IL‐24 activity at 50–150 ng/mL in cell culture systems, particularly 75–125 ng/mL for fibroblast applications.^7^, ^8^ Time‐lapse microscopy confirmed significant cellular expansion following recombinant IL‐24 (rIL‐24) administration (Figure 2B and C). Flow cytometric analysis revealed elevated cell populations in G1 and G2 phases after rIL‐24 treatment, confirming enhanced proliferative capacity (Figure 2D and E). Wound healing assays demonstrated substantially augmented migratory potential in rIL‐24‐treated fibroblasts (Figure 2F and G). Western blot analysis showed markedly upregulated expression of α‐SMA and COL1 in response to rIL‐24, indicating enhanced myofibroblast differentiation and collagen production (Figure 2H–K). These findings collectively establish that IL‐24 remarkably boosts core fibroblast capabilities crucial for wound recovery, specifically proliferation, migration, and FMT.

In this study, we identified GCDs from Northeast China ginseng as an effective antibacterial agent. GCDs synthesised through 8‐h solvothermal treatment at 200°C demonstrated robust photoluminescence and exceptional fluorescent properties (Figure 3A). HRTEM revealed uniform morphology without aggregation, with approximately 10 nm diameter, confirmed by particle size analysis (Figure 3B and C). The negative zeta potential indicated capacity to attract positively charged bacteria (Figure 3D). XPS and FTIR analyses confirmed C, N, and O as primary constituents, with characteristic vibration peaks at 1750, 2854, and 3400 cm^−1^ (Figure 3E–I). CFU assays demonstrated significant inhibition of both *S. aureus* and *P. aeruginosa* proliferation (Figure 3J–L). Live/Dead staining verified bacterial death, establishing broad‐spectrum antimicrobial efficacy (Figure 3M–O). Flow cytometry revealed GCDs substantially enhanced ROS production in both bacterial strains, elucidating their bactericidal mechanism through oxidative stress (Figure 3P–R). The ROS released by GCDs cause negligible damage to normal tissue cells. The differential effects on bacterial versus tissue cells can be attributed to fundamental differences in cellular antioxidant capacity. Mammalian cells possess sophisticated enzymatic defence systems (SOD, catalase, GPx) and compartmentalised organelles for ROS scavenging, while bacterial antioxidant systems are primitive and limited, rendering them more susceptible to oxidative damage. Moreover, GCDs exhibit cytoprotective properties by activating endogenous antioxidant pathways upon cellular uptake, further enhancing the protective effects in tissue cells.^9^, ^10^ We employed thermosensitive hydrogel pluronics (HGP) as the delivery vehicle for rIL‐24 and GCDs. HGP exhibits excellent fluidity at room temperature while solidifying at body temperature, enabling precise adaptation to diverse wound topographies (Figure S1A and B). This biocompatible carrier achieves sustained rIL‐24 release over five days, confirmed through hemolysis tests showing PBS‐equivalent safety profiles, with the hydrogel matrix showing controlled biodegradation (Figure S1C–F). To evaluate our composite material's therapeutic efficacy on diabetic wounds, we established mouse models treated with HGP (control), HGP@GCDs, or HGP@rIL‐24&GCDs (Figure 4A). H&E staining of major organs confirmed excellent biocompatibility across all formulations, with no detectable abnormalities (Figure 4O). The therapeutic assessment revealed HGP@rIL‐24&GCDs significantly accelerated wound closure compared to both control and HGP@GCDs groups (Figure 4B–D). Histological analysis through H&E staining demonstrated significantly reduced wound widths in the HGP@rIL‐24&GCDs group (Figure 4E and F). Masson staining exhibited more organised collagen fibre architecture and elevated fibrosis rates following HGP@rIL‐24&GCDs treatment (Figure 4G and H). Though GCDs' antibacterial properties modestly enhanced healing, IL‐24 emerged as the pivotal therapeutic component. Further investigations revealed HGP@rIL‐24&GCDs substantially augmented fibroblast proliferation, myofibroblast differentiation, and collagen synthesis compared to alternative treatments, underscoring IL‐24′s crucial role in diabetic wound repair mechanisms (Figure 4I–N). Overall, our research identified IL‐24′s critical role in diabetic wound healing through FMT induction, confirmed via knockout studies. We developed an innovative asymmetric dual‐layer hydrogel dressing: the tissue‐facing layer delivers IL‐24 to enhance tissue regeneration, while the external layer incorporates GCDs that release ROS for antimicrobial protection. Animal models validated this dressing's dual functionality in accelerating wound closure and preventing infection. This spatiotemporal approach effectively addresses bacterial vulnerability while maximising IL‐24′s regenerative capacity, representing a promising comprehensive strategy for improved diabetic wound management. Although limitations include single diabetic model validation, short‐term assessment, and incomplete molecular mechanism characterisation, our thermosensitive hydrogel platform demonstrates exceptional clinical translation potential with practical application advantages and excellent biocompatibility. The dual‐functionality approach addresses critical unmet clinical needs, requiring only standardised manufacturing and clinical validation for successful therapeutic implementation. In conclusion, this study provides compelling evidence for IL‐24′s therapeutic potential in diabetic wound healing and demonstrates the feasibility of an innovative dual‐layer delivery system. While challenges remain for clinical translation, the fundamental scientific advances and therapeutic strategy presented here offer significant promise for addressing the unmet clinical need in diabetic wound management.

**FIGURE 2:**
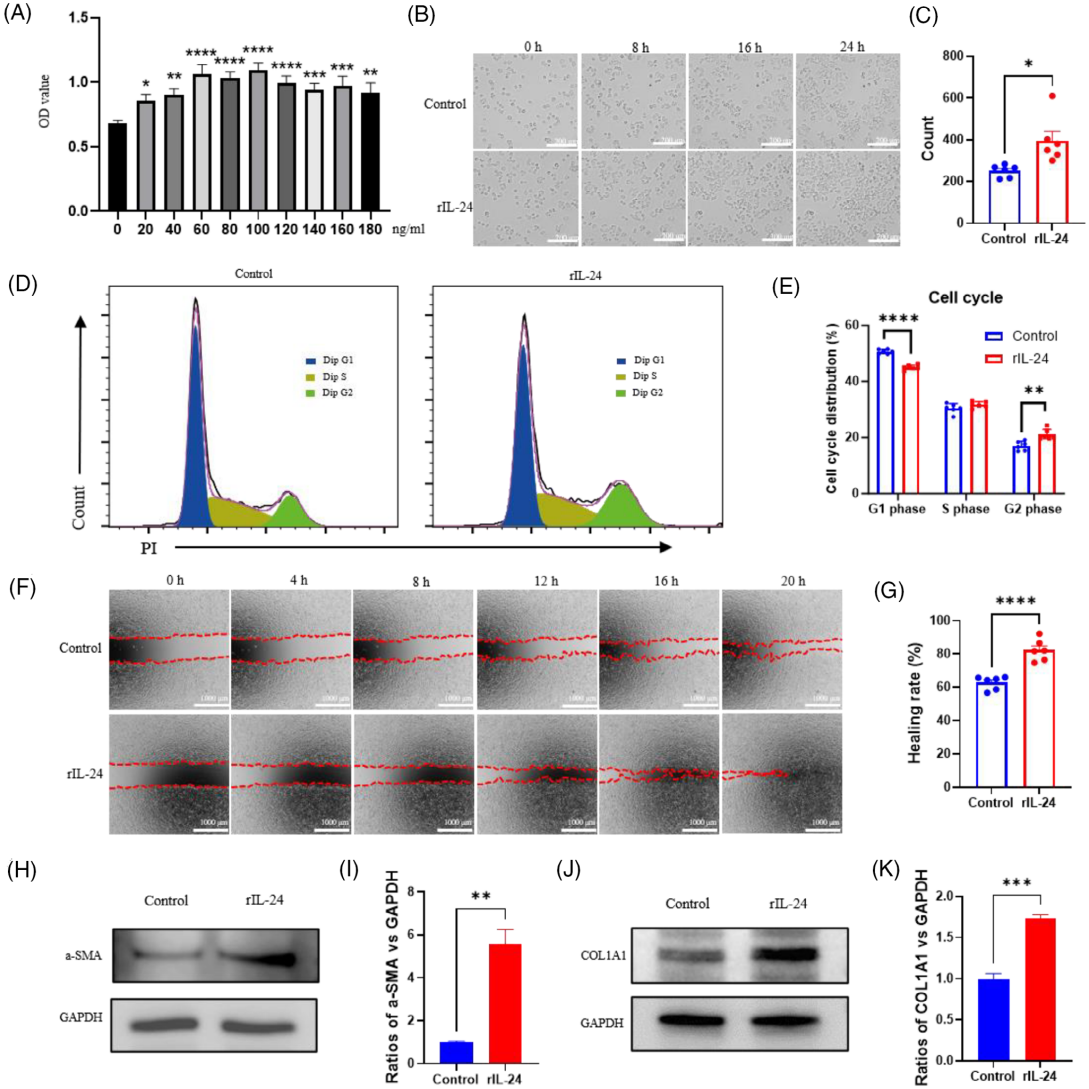
rIL‐24 accelerated fibroblast proliferation and migration. (A) CCK‐8 assays of fibroblasts (L929 cells) treated with rIL‐24 in various concentrations from 0 to 180 ng/mL. (B, C) Living cell analysis of fibroblasts treated with rIL‐24 (100 ng/mL). (*n* = 6 per group.) (D, E) Cell cycle assessment of fibroblasts treated with rIL‐24 (100 ng/mL) by flow cytometry. (*n* = 6 per group.) (F, G) Wound healing assays of fibroblasts treated with 100 ng/mL rIL‐24. (*n* = 6 per group.) (H, I) The protein expression levels of α‐SMA in fibroblasts treated without and with 100 ng/mL rIL‐24. (*n* = 3 per group.) (J, K) The protein expression levels of COL1A1 in fibroblasts treated without and with 100 ng/mL rIL‐24. (*n* = 3 per group.) *p < .05, **p < .01, ***p < .001, ****p < .0001, one‐way ANOVA followed by Bonferroni's multiple comparison test. All values are presented as means ± SEM.

**FIGURE 3:**
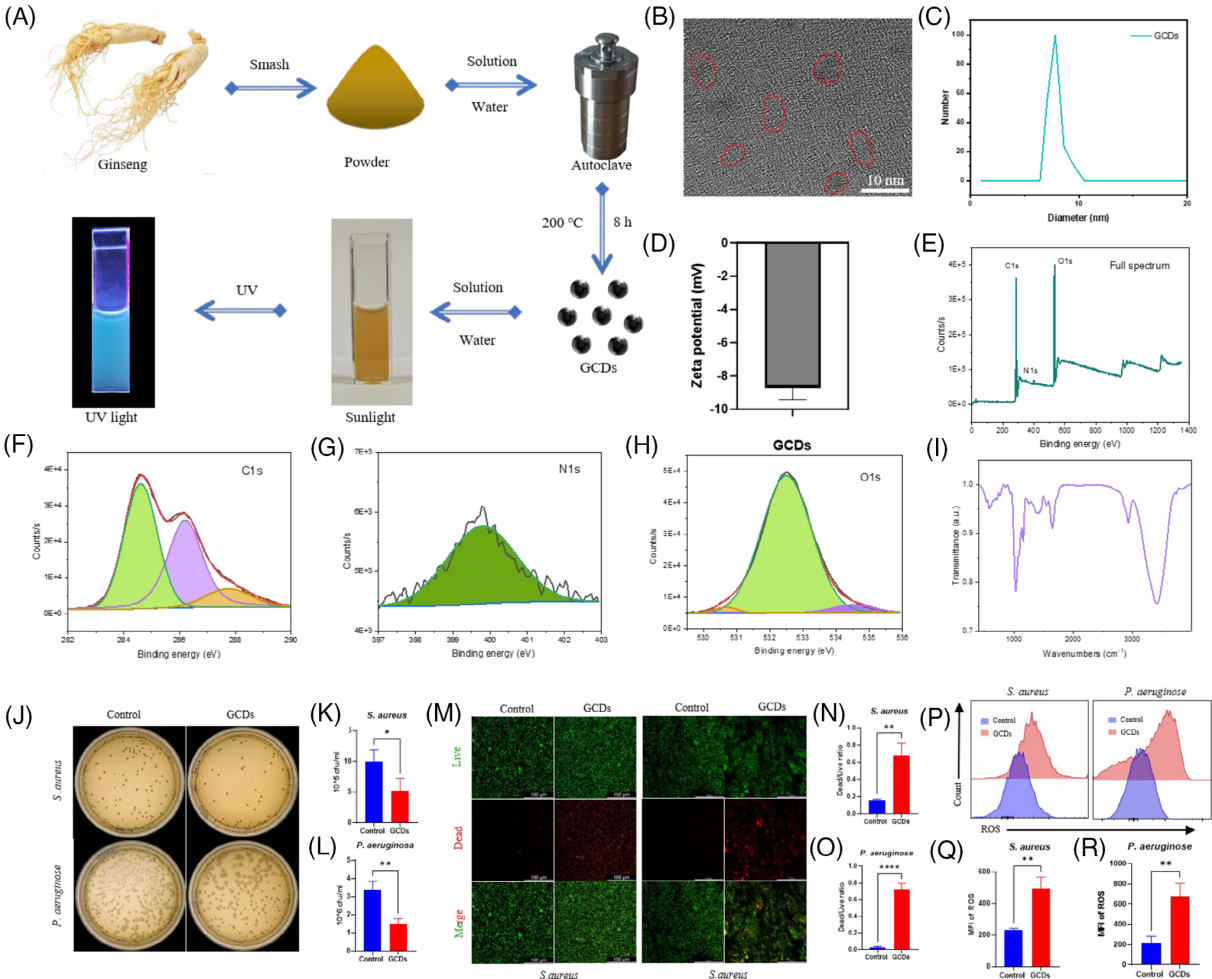
Synthesis, characterisation, antibacterial properties of GCDs. (A) Schematic diagram of the GCDs synthesis workflow. (B) HRTEM image of GCDs. (C) Analysis of particle size of GCDs. (D) The zeta potential of GCDs. (*n* = 3 per group.) (E–H) XPS spectra of GCDs. Binding energy of GCDs in full spectrum(E), C1s(F), N1s(G), O1s(H). (I) The Fourier infrared spectrum diagram of GCDs. (J–L) The colony count of *S. aureus* and *P. aeruginosa* treated with GCDs. (*n* = 3 per group.) (M–O) The Living‐dead staining showing the antibacterial properties of GCDs on *S. aureus* and *P. aeruginosa*. (*n* = 3 per group.) (P–R) The ROS production of *S. aureus* and *P. aeruginosa* treated with GCDs by flow cytometry. (*n* = 3 per group.) *p < .05, **p < .01, one‐way ANOVA followed by Bonferroni's multiple comparison test. All values are presented as means ± SEM.

**FIGURE 4:**
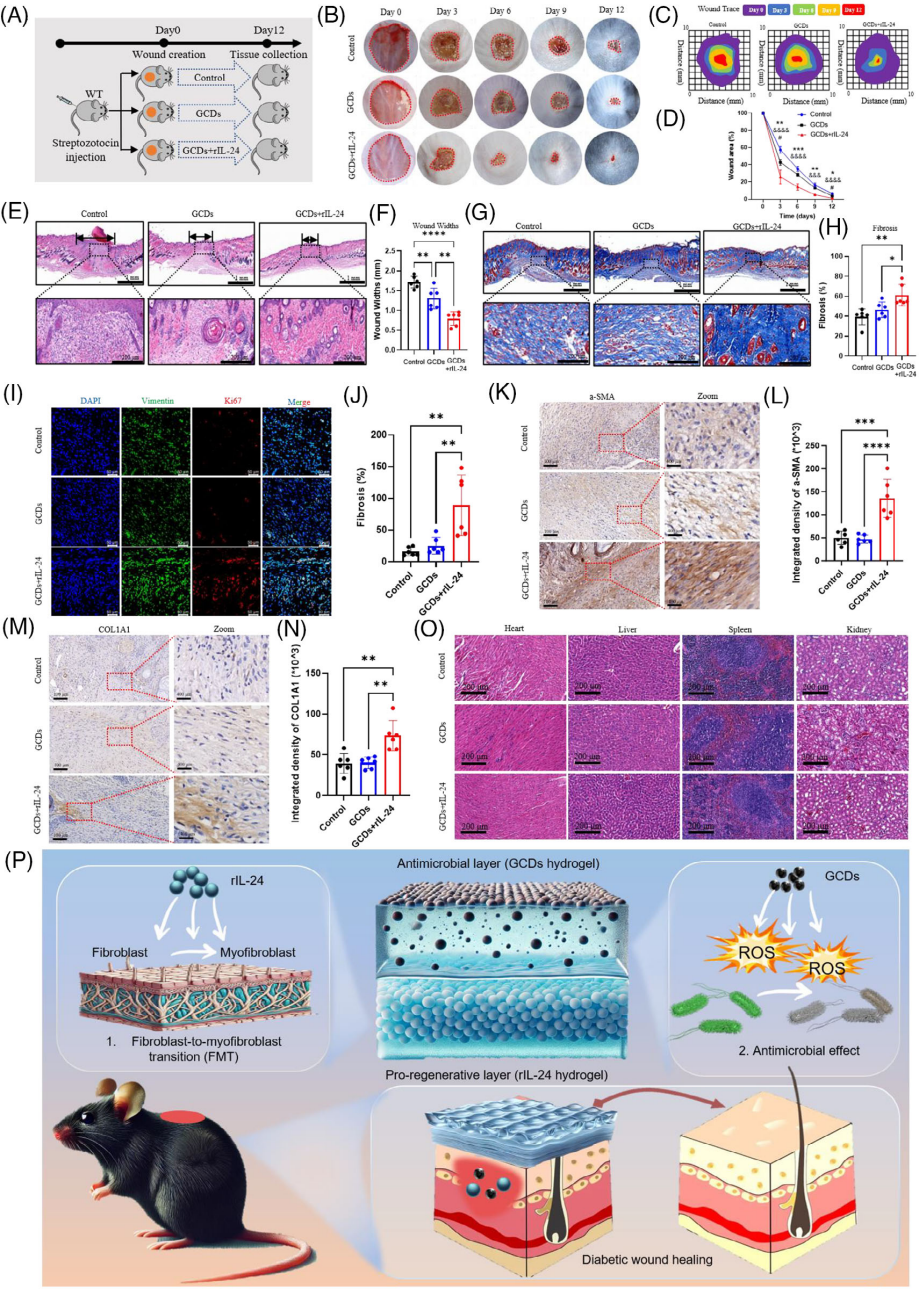
rIL‐24 combination with GCDs promoted the healing of diabetic wounds. (A) Schematic diagram of the diabetic wound construction treated with PBS (control), HGP loaded with GCDs (GCDs) and HGP loaded with rIL‐24 and GCDs (GCDs+rIL‐24). (B) Images showing morphological changes of the diabetic wounds. (C) The wound trace of the diabetic wounds. (D) The wound area ratio of the diabetic wounds. (*: the statistical differences between the GCDs+ rIL‐24 group the GCDs group; &: statistical variations evaluated upon comparing the GCDs+ rIL‐24 group against the control group; #: statistical variations evaluated upon comparing the GCDs group against the control group. *n* = 6 per group.) (E, F) H&E staining showing changes of the diabetic wounds. (*n* = 6 per group.) (G, H) Masson staining showing the changes of fibrosis area. (*n* = 6 per group.) (I, J) IF staining demonstrating the expression of Vimentin and Ki67 in the diabetic wounds. (*n* = 6 per group.) (K, L) IHC detection demonstrating the distribution of α‐SMA. (*n* = 6 per group.) (M, N) IHC staining showing the expression of COL1A1. (*n* = 6 per group.) (O) HE staining showing the anatomical structure of hearts, livers, spleens and kidneys in diabetic wounds mice of different groups. (P) A schematic diagram of the asymmetric double‐layer dressing loaded with IL‐24 and GCDs for diabetic wound healing interventions. *or #*p* < .05, **p < .01, *** or &&&*p* <.001; &&&&*p* < .0001, one‐way ANOVA followed by Bonferroni's multiple comparison test. All values are presented as means ± SEM.

## AUTHOR CONTRIBUTIONS

Conceptualisation: W.X. Investigation: S.L., J.L., N.J. Formal analysis: N.J., S.H. Writing: S.L., J.L. Funding acquisition: W.X. Supervision: Y.S., J.H.

## CONFLICT OF INTEREST STATEMENT

The authors declare that they have no known competing financial interests or personal relationships that could have appeared to influence the work reported in this paper.

## ETHICS STATEMENT

The animal welfare and experimental procedures in this study were approved by the Animal Care and Use Committee of Ruige Biotechnology (No.: 20240410‐003).

## Supporting information



Supporting Information

## Data Availability

The data that support the findings of this study are available from the corresponding author upon reasonable request.
