# Supplementary material for: Asymmetric bilayer dressings with spatiotemporal sequence loaded with IL‐24 and GCDs for the treatment of diabetic wounds
Source: Clin Transl Med. 2025 Jul 16;15(7):e70402. doi: 10.1002/ctm2.70402 (PMC12267660; doi:10.1002/ctm2.70402)
Supplement: Supplementary file 1 — Supporting Information [file CTM2-15-e70402-s001.docx]

# Supporting Information

Asymmetric bilayer dressings with spatiotemporal sequence loaded with IL-24 and GCDs for the treatment of diabetic wounds

Sijia Li^a, 1^, Jinjin Lu^c, 1^, Nianqiang Jin^b, 1^, Yuan Su^d^, Songning Han^e^, Jiankang He^b, *^, Wenqiang Xie^b, *^

^a^ Foshan Stomatology Hospital & School of Medicine, Foshan University, Foshan, 528000, PR China.

^b^ Stomatological Hospital, Southern Medical University, Guangzhou 510280, PR China.

^c^ Department of Periodontics, Affiliated Stomatology Hospital of Guangzhou Medical University, Guangzhou Key Laboratory of Basic and Applied Research of Oral Regenerative Medicine, Guangzhou, 510182, PR China.

^d^ Department of Periodontology, Stomatological Hospital, Southern Medical University, Guangzhou, China; Stomatology Center, Shunde Hospital, Southern Medical University (The First People’s Hospital of Shunde), Foshan, 510280, PR China.

^e^ School of Stomatology, Jinan University, Guangzhou, 510182, PR China.

^*^ Corresponding authors.

*E-mail addresses:* xwq_smu@foxmail.com

^1^ *These authors (S.* L., *J. L. and N.J.) contribute equally to this work.*

**Figure S1**. Characterization and biocompatibility of HGP.

# Methods

**Animals**

The animal welfare and experimental procedures in this study were approved by the Animal Care and Use Committee of Ruige Biotechnology. (No.: 20240410-003). All mice were in C57BL/6J background and were cultured under specific pathogen free (SPF) conditions. The wild-type (WT) mice were purchased from GemPharmatech Co., Ltd, while the IL-24-/- knockout (KO) mice (#S-KO-15440) were generated by Cyagen biological Co., Ltd (China, Suzhou). In brief, the IL-24 gene (NCBI Reference Sequence: NM_053095.2; Ensembl: ENSMUSG00000026420) is located on mouse chromosome 1. Six exons are identified, with the ATG start codon in exon 1 and the TGA stop codon in exon 6 (Transcript Il24-201: ENSMUST00000121040). Exon 2~5 was selected as target site. Following the CRISPR/Cas mediated genome editing procedures, sgRNA was designed to high-throughput electroporation of fertilized eggs to obtain IL-24 KO heterozygous mice (IL-24-/+). The offspring of IL-24-/+ mice were genetically identified by PCR to obtain IL24 KO mice (IL-24-/-). 4-week-old IL-24-/- KO mice were used for subsequent experiments.

**Bioinformatic analysis and verification of IL-24 gene expression in mouse diabetic wounds**

Mouse diabetic wound chip data was obtained from GEO database (GSE147890). Differentially expressed genes (DEGs) were screened through GEO2R and processed for analysis. Volcano plots, violin plots, and heatmaps of differentially expressed genes were constructed for the IL-24 gene and wound healing-related genes. Diabetic wound models were established to verify the differential expression of IL-24 and its relationship with diabetic wound healing.

**Establishment of skin wound model in diabetic mice**

Diabetic models were stimulated in 4-week-old c57BL/6J mice via administering 50 mg/kg streptozotocin (STZ) intraperitoneally. Blood glucose measurements were performed via tail vein glucometer (Yuwell, China) a week post-STZ injection, and readings exceeding 16.7 mmol/L confirmed diabetes establishment. After dorsal hair removal, complete skin defects (8 mm in diameter) were generated. According to the group requirements, the hydrogel materials were applied to the wounds. Wound healing was monitored through digital imaging and quantified using Image J analysis at 3-day intervals until day 12. On day 12, after euthanasia, peri-wound tissues were harvested and preserved in 4% paraformaldehyde for further analysis.

**Pathological staining of wound tissue**

The wound tissues fixed with paraformaldehyde were embedded, and 4 µm thick sections were cut for H&E staining and Masson staining. Data were collected using a pathological scanner (Aperio VERSA, Leica Biosystems, Germany). The staining data were analyzed using Image J software.

**Immunohistochemical staining of wound tissue**

An overnight immunostaining protocol at 4°C was conducted on the tissue sections using primary antibodies targeting alpha-SMA (ab18147, Abcam, UK) and COL1A1 (ab90395, Abcam, UK). Then, secondary antibody conjugated with horseradish peroxidase (HRP, Abcam, UK) was used to treat the samples for 1 hour at 37 degrees Celsiusthey, followed by washing. DAPI (Invitrogen, USA) was used for counterstaining to observe the cell nuclei. Visualization of the labeled cells was performed using an Olympus BX51 microscope, while maintaining identical settings for image acquisition with an Olympus DP70 digital camera. ImageJ software was used for analysis.

**Immunofluorescence staining of wound tissue**

Sections were probed using primary antibodies anti-Ki67 (ab66155, Abcam, UK) and Vimentin (Abcam, UK) 4 °C overnight. Following PBS washing, samples were incubated with Alexa Fluor 488/594-conjugated donkey secondary antibodies (Invitrogen, USA) for 1.5 hours at room temperature. Nuclear counterstaining was performed with DAPI before confocal laser microscopic (Leica Instruments GmbH, Germany) analysis and imaging. The analysis was performed using ImageJ software.

**The CCK-8 assay for detecting the impact of IL-24 on the proliferation of L929 cells**

L929 cells were continuously cultured *in vitro* in high-glucose medium (4.5 g/L glucose, approximately 25 mM) to simulate the hyperglycemic environment *in vivo*. L929 cells, plated in 96-well format, were exposed to multiple concentrations of rIL-24 protein (0-180 ng/mL). Fresh medium (100 μL) and cell counting kit-8 (CCK-8) reagent (10% v/v) (Yeasen Biotechnology Co., Ltd., China) were introduced to the cultures after 24 hours of incubation. After 120 minutes of incubation, a microplate reader (Agilent Technologies, USA) was used to measure cell viability at 450 nm.

**Live-cell imaging system for detecting the effects of IL-24 on the proliferation and migration of L929 cells**

Proliferation: Using a live cell imaging system, cellular proliferation was monitored. The experimental setup involved seeding resuspended L929 cells into 96-well plates at 3000 cells/well. The IncuCyte ZOOM imaging system (Essen Bioscience, USA), which is used for real-time assessment of cell confluence, took photos every 4 hours to record cell growth.

Migration: A cell suspension of L929 cells was prepared and seeded into 24-well plates, achieving a final density of 3×10^4^ cells/well. The experiment was conducted when the cells adhered and reached a density of 80% to 90%. Using a sterile 200 μL pipette tip, vertical scratches were created in each well. After gentle PBS washing to remove debris and detached cells, the cultures were exposed to varying concentrations of recombinant IL-24 protein. Cell migration was monitored using the IncuCyte ZOOM system (Essen Bioscience, USA), with images captured at 4-hour intervals. Migration rates were determined by analyzing the scratch area changes between initial and final timepoints.

**Western blot assay**

Protein concentrations were determined using a Pierce Bis-Tris Acrylamide (BCA) Protein Quantification Assay Kit (Thermo Fisher Scientific, USA). Following separation on 12% SDS-PAGE gels, equivalent protein amounts were transferred to PVDF membranes (Millipore, USA). The membranes underwent blocking with 5% non-fat milk at 4 °C, followed by overnight incubation with ASMA and COL1A1 primary antibodies at 4°C. After exposure to specific secondary antibodies for 2 hours at 37 °C, protein bands were detected using a gel imaging system (Shanghai Tianeng Life Sciences Co., Ltd., China).

**Flow cytometry for detecting the impact of IL-24 on the cell cycle of L929 cells**

Trypsin-treated L929 cells were collected and underwent fixation in 70% ethanol (24 hours, 4 °C). The fixed cells were washed with PBS before incubation with 500 μL propidium iodide (PI) (Beyotime Biotechnology, China) at 37 °C for 30 minutes. A flow cytometer (Beckman Coulter Inc., USA) was used to quantify cell cycle phases, followed by FlowJo analysis.

**Preparation and characterization of ginseng carbon quantum dots**

Preparation: GCDs were synthesized using a solvothermal procedure. The ginseng was ground into powder using a micro-grinding machine, and 1.0 g of the powder was added to 10 mL of water and thoroughly mixed. The mixture was sealed in a 20 mL polytetrafluoroethylene (PTFE) Teflon-lined autoclave and maintained at 200°C for 8 hours. Upon cooling to ambient temperature, the solution underwent centrifugation (8000 r/min for 10 minutes). The resulting dark yellow supernatant was passed through a 0.22 μm polyethersulfone filter to eliminate larger particles. The GCD solution was then subjected to 24-hour dialysis in deionized water to remove residual reactants and small molecular by-products. Finally, the purified aqueous solution of GCDs was condensed in a vacuum rotary evaporator and re-dissolved in deionized water for future use.

Characterization: The fourier transform infrared (FTIR) of GCDs were investigated by fourier transform infrared spectrometer (Thermo Scientific, USA). The chemical composition of GCDs was measured by X-ray photoelectron spectroscopy (XPS) instrument (Kratos, UK) utilizing a monochromatic aluminum Kα X-ray source. Particle size and zeta potential of GCDs were measured using a particle size analyzer (Brookhaven Instruments Corporation, USA).

**Preparation of the hydrogel**

The dosage ratio of F127 to F68 is 26% w/w and 4% w/w respectively. F127 and F68 are weighed in a ratio of 26% w/v to 4% w/v. The weighed F68 is dissolved in deionized water (NS) at 4 ℃ to prepare a solution. Then, F127 is dissolved in the solution at 4 ℃ and thoroughly mixed and shaken to form a physical cross-linked hydrogel. This hydrogel is stored in a 4 ℃ refrigerator for future use.

**Characterization of hydrogel properties**

Thermal sensitivity performance: Prepare hydrogels. When the temperature drops to 4 ℃, invert the sol and observe its flow condition. Then place it in a 70 ℃ water bath and invert it every 30 seconds to observe its flow condition and gelation time. Injectability: Prepare hydrogels. When the temperature drops to 4 °C, then inject the hydrogel with a 1 mL syringe. For degradation assessment, 1 mL of hydrogel was placed at the bottom of centrifuge tubes and allowed to solidify at 37 °C. The solidified hydrogel was then immersed in 5 mL PBS and maintained at 37 °C. Weight measurements were recorded at predetermined time intervals to calculate the degradation rate.

**Biocompatibility testing of hydrogel materials**

Hemolysis test: Place the hydrogel in a centrifuge tube. At 37 °C with gentle stirring, add 200 µL of diluted blood (200 µL of EDTA-killed whole blood added to 10 ml of 0.9% saline and mixed with the test sample tube for 2 hours) to quantify the hemolysis caused by the hydrogel material. The anticoagulant blood was mixed with Trizol and physiological saline respectively, serving as positive and negative controls. Blood compatibility was quantitatively assessed through hemolysis rate calculations. Following 10-minute centrifugation at 1000 rpm, supernatant absorbance was measured at 540 nm using a spectrophotometer plate reader. The measurements were performed in triplicate, with mean values used for final calculations using the designated formula: Hemolysis rate (%) = (A sample - A negative) / (A positive - A negative) × 100%.

**Experiment on antibacterial properties of hydrogel prepared with ginseng carbon quantum dots**

CFU Counting Experiment: The inhibition activity was determined by the coating method. Escherichia coli and Pseudomonas aeruginosa were used as materials to test the surface inhibition activity. Take the ultraviolet-sterilized freeze-dried hydrogel material, place it in a 5 mL centrifuge tube, add 3 mL bacterial culture medium to prepare the extract (6.67 mg/mL); add 50 μL of Escherichia coli or Pseudomonas aeruginosa to the extract without adding the material as the blank control group. After sealing, it was cultured in a 37 ℃, 100 rpm shaking incubator for 6 h, then diluted in multiples, and each sample was spread on 3 parallel plates after sealing. After culturing at 37 ℃ for 18 hours, count the colony-forming units (CFU) on the culture plates.

Flow cytometry for bacterial viability staining: LIVE/DEAD BacLight components (PI, SYTO9; Invitrogen, USA) and SYBR Green were diluted 100-fold in sterile-filtered DMSO and maintained at −20°C. For bacterial staining, samples were treated with SYTO9/PI mixture (5 μM/30 μM) and incubated in darkness at ambient temperature (20-25 minutes). EDTA treatment (5 mM, pH 8) was used for membrane permeabilization. Analysis was performed on a flow cytometer (Beckman Coulter, USA) using 488 nm excitation (50 mW blue laser), with fluorescence detected at FL1 (520 nm, green) and FL3 (630 nm, red). The FL1 channel served as the trigger.

Confocal laser scanning microscopy for bacterial viability staining: After the same staining operation on bacterial samples, a confocal laser scanning microscope (Leica Instruments GmbH, Germany) was used to visualize and capture images of the fluorescent staining, which were analyzed by Image J software.

**Flow cytometry for investigating the effect of hydrogel containing luteinized carbon quantum dots on bacterial ROS**

ROS levels in bacterial samples were evaluated using 2',7'-dichlorofluorescein diacetate (DCFH-DA, Beyotime, China), an intracellular ROS probe. Following exposure to carbon quantum dots hydrogel, samples were stained with DCFH-DA (10 μM) and incubated in darkness at 37 °C for 20 minutes. After PBS washing to eliminate excess dye, the labeled cells were analyzed by flow cytometry.

**Statistical analysis**

Data analysis was performed using GraphPad Prism software. Results are presented as mean ± SEM from independent experimental replicates. For comparisons between two groups, unpaired two-tailed Student's t-tests were employed. One-way ANOVA was utilized for comparing three or more groups with a single variable. Significance levels were denoted as: ns (not significant), ∗ (p < 0.05), ∗∗ (p < 0.01), ∗∗∗ (p < 0.001), and ∗∗∗∗ (p < 0.0001). Detailed statistical methods are provided in figure legends.

**Figure S1**


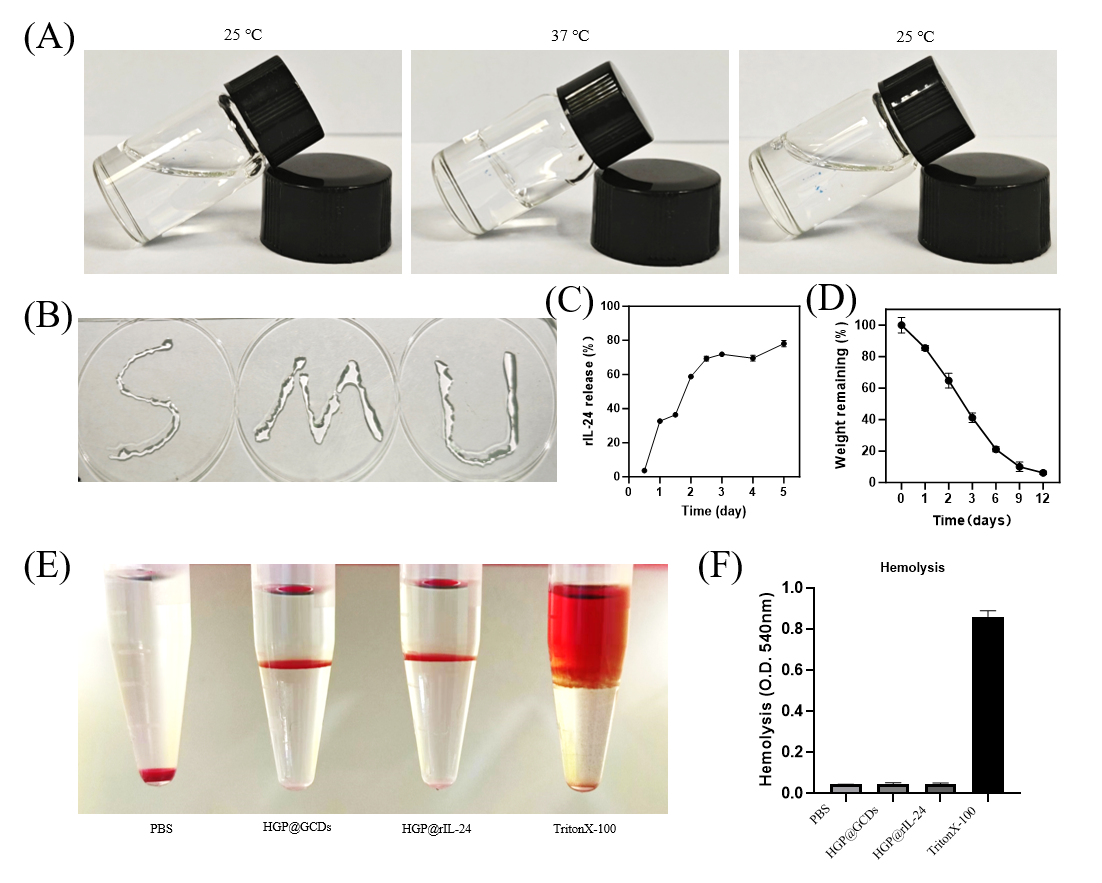


**FIGURE S1.** Characterization and biocompatibility of HGP. (A) The state transfer images of thermosensitive hydrogel of HGP at a continuous temperature change (25 °C→37 °C→25 °C). (B) HGP formed “SMU”. (C) Slow-release performance of HGP hydrogel loaded with rIL-24. (D) The degradation performance of HGP hydrogel. (E, F) Hemolysis assays of PBS, HGP loaded with GCDs, HGP loaded with rIL-24 and PBS with TritonX-100.
